# Embedded cell-only bioprinting to engineer structurally aligned meniscal fibrocartilage

**DOI:** 10.1016/j.mtbio.2026.103308

**Published:** 2026-06-11

**Authors:** Aliaa Sherif Karam, Gabriela S. Kronemberger, Kaoutar Chattahy, Diana Eveline Sanchez-Amador, Michael G. Monaghan, Daniel J. Kelly

**Affiliations:** a Trinity Centre for Biomedical Engineering, Trinity Biomedical Sciences Institute, Trinity College Dublin, Dublin, D02 PN40, Ireland; b Discipline of Mechanical, Manufacturing and Biomedical Engineering, School of Engineering, Trinity College Dublin, Dublin, Ireland; c Advanced Materials and Bioengineering Research Centre (AMBER), Royal College of Surgeons in Ireland and Trinity College Dublin, Dublin, Ireland; d CÚRAM, Centre for Research in Medical Devices, National University of Ireland, Galway, H91 W2TY, Ireland; e Department of Anatomy and Regenerative Medicine, Royal College of Surgeons in Ireland, Dublin, Ireland

**Keywords:** Meniscus, Embedded bioprinting, Collagen alignment, Boundary conditions

## Abstract

The engineering of functional meniscal grafts remains elusive, largely due to an inability to recapitulate the highly organized collagen architecture of the native tissue which is integral to its biomechanical function. In this study, we investigated whether external geometric confinement can direct collagen alignment in fibrocartilaginous tissues generated by mesenchymal stem/stromal cells (MSCs). First, MSCs were cast within non-adhesive agarose channels, which supported the development of fibrocartilaginous tissues with collagen fibres aligned parallel to the long axis of the confining agarose wells. To translate these findings into a scalable biofabrication platform, MSC-only bioinks were bioprinted into a methacrylated xanthan gum (XGMA) support bath to generate filaments of differing widths. It was found that reducing filament width enhanced collagen alignment and fibrocartilaginous matrix deposition. To elucidate the role of cellular mechanotransduction on the observed boundary induced collagen organization, YAP and ROCK pathways were inhibited during culture. While inhibition disrupted cytoskeletal and nuclear alignment, collagen organization remained highly aligned, with no observed differences in Brillouin frequency shift. Finally, this strategy was scaled to fabricate anisotropic fibrocartilage sheets and circumferentially organized meniscus-like constructs. Overall, the findings of this study establish external geometric confinement as a powerful and scalable strategy to engineer meniscal grafts with a more biomimetic collagen organization, which may pave the way for the future development of scaffold-free meniscal grafts.

## Graphical abstract

**Figure:**
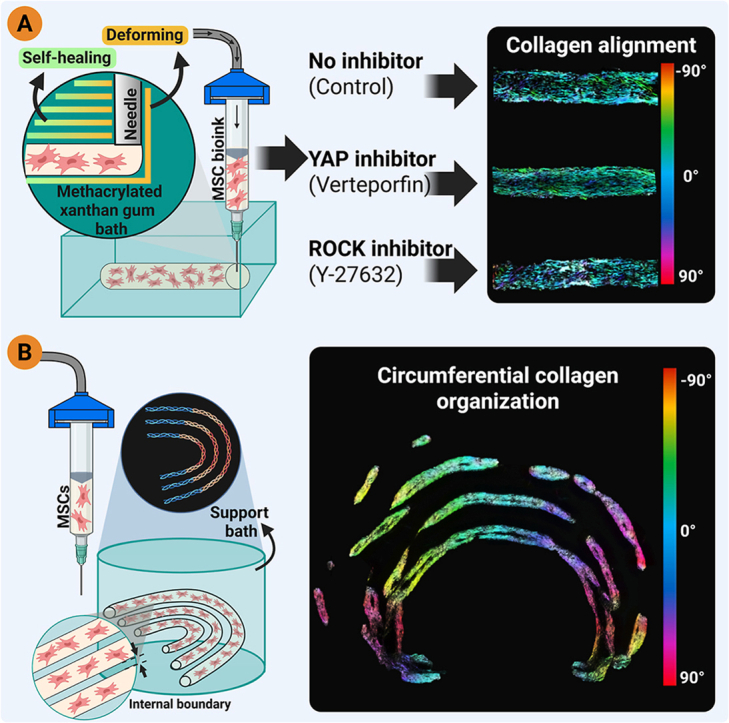

## 1 Introduction

Collagen organization within musculoskeletal tissues is integral to their biomechanical function. For example, during articular cartilage development it has been shown that postnatal changes to the collagen organization determine the mechanical properties of the tissue [1]. This is also evident in the knee meniscus, a wedge-shaped fibrocartilaginous tissue that acts as a shock absorber and protects the underlying articular cartilage [2]. The meniscus is characterized by a highly organized collagen architecture, dominated by circumferentially aligned collagen fibers that resist tensile stresses and enable efficient load distribution [3]. In addition, radially oriented collagen fibres, often referred to as tie fibres, stabilize the circumferential network by resisting radial strains [3]. Damage to the meniscus resulting from injury or age-related degeneration is highly prevalent where, for example, the prevalence of knee meniscal damage is 35% in the general population of middle aged and older individuals [4,5]. Additionally, meniscus damage significantly increases the risk of developing osteoarthritis, a debilitating and painful inflammatory joint disease [6,7]. Current treatment options for severe meniscal lesions or tears, including partial or complete meniscectomy, provide only temporary pain relief and are associated with reduced tissue functionality, altered joint biomechanics, and increased risk of osteoarthritis [8,9]. To date, classical tissue engineering approaches have failed to reproduce the complex, biomimetic collagen architecture of the native meniscus, resulting in constructs with limited mechanical performance [10,11]. Consequently, there remains an unmet clinical need for engineered meniscal grafts within biomimetic collagen network organization and hence functional biomechanical properties [12,13].

Three-dimensional (3D) bioprinting has emerged as a promising biofabrication technique for meniscus engineering, as accurate control over implant size and anatomical matching are critical determinants of meniscal transplant outcomes [[14], [15], [16], [17]]. Although several studies have reported bioprinting of meniscal constructs, many have focused primarily on supporting fibrochondrogenesis and matching the macroscopic geometry of the native tissue, with limited assessment of collagen alignment within the engineered construct [[18], [19], [20], [21], [22], [23]]. Recognizing the importance of cell and matrix organization for functional meniscus regeneration, varying bioprinting strategies have been developed to guide not only implant geometry but also collagen architecture [24,25]. For example, melt electrowritten (MEW) polycaprolactone (PCL) scaffolds have been used as physical boundaries to direct the alignment of neotissue collagen secreted by inkjet bioprinted cells [24], while incorporating hyaluronic acid-based microfibers into bioinks that align during extrusion bioprinting has been shown to guide cellular orientation and neotissue collagen deposition [25]. Despite their effectiveness, microfiber additives can increase bioink viscosity and the risk of nozzle clogging during printing. More recently, embedded bioprinting has emerged as a promising strategy to impose physical boundary condition cues without modifying bioink composition [26,27]. We previously demonstrated this concept using articular cartilage progenitor cells bioprinted in a methacrylated xanthan gum (XGMA) support bath, where strategically designed boundary conditions served as geometric cues that directed cell alignment and matrix organization along the long axis of printed constructs [26]. However, the application of XGMA support baths to meniscus tissue engineering, as well as the mechanisms governing boundary-induced collagen alignment in this context, remain unexplored.

Following secretion of procollagen, the collagen precursor molecule, fibrillogenesis occurs within the extracellular space, where molecular crowding, geometric constraints and physical confinement are known to influence collagen alignment and matrix organization [26,[28], [29], [30], [31]]. While we and others have demonstrated that physical confinement of cells or microtissues can guide neotissue collagen alignment *in vitro* [26,27,30], the mechanisms underlying this phenomenon remain largely unexplored. It is well known that through the activation of various mechanotransduction pathways, cells can sense and respond to their surrounding external environment. These include Yes-associated protein (YAP) and Rho-associated kinase (ROCK) signalling pathways which enable cells to reorganize their cytoskeleton, generate forces and remodel their extracellular matrix (ECM) [[32], [33], [34], [35], [36], [37], [38]]. Specifically, ROCK enables cell-matrix adhesion, stress fibre formation and cell contractility by phosphorylation of myosin light chain and involvement in focal adhesion assembly [[38], [39], [40], [41], [42]]. YAP is a mechanosensitive transcription factor that translocates into the nucleus upon activation, where it regulates the expression of different genes involved in cell differentiation, contraction, cytoskeletal and ECM remodelling [32,33]. In the context of cell and collagen alignment, it is unclear whether boundary-induced effects arise predominantly from such active, cell-mediated responses to physical confinement or from passive, boundary-driven (re-)organization of the ECM independent of cell-generated forces. Therefore, the aims of this study were to **(1)** investigate the effect of XGMA boundary spacing on the alignment and organization of bioprinted mesenchymal stem/stromal cells (MSCs), **(2)** elucidate the role of cellular mechanotransduction and contractility in boundary-induced collagen alignment by inhibiting key signalling pathways, including YAP and ROCK, and **(3)** bioprint a fibrocartilage tissue with a circumferential collagen network architecture, thereby enabling the engineering of more biomimetic meniscal grafts.

## 2 Material and methods

### 2.1 Isolation and expansion of MSCs

MSCs were isolated from the bone marrow of the sternum from skeletally mature female goats. The bone marrow was chopped to around 3 mm^3^ pieces and placed in expansion media (XPAN) composed of high-glucose DMEM (Bioscience, Ireland) supplemented with 10% (v/v) fetal bovine serum (FBS, Gibco, Ireland), 100 U/mL penicillin, 100 μg/mL streptomycin, and 2.5 μg/mL amphotericin B (Sigma, Ireland) and then vortexed to liberate the cells. The solution was sieved to remove any pieces of bone and other debris. The MSCs were cultured at 5% O_2_ and 5% CO_2_ stimulation and trypsinized once colonies were 80% confluent and expanded until passage 4.

### 2.2 Fabrication of agarose boundaries and bioink casting

Autoclavable positive moulds containing 12 mm long, 1 mm high and 500 μm wide cuboids were 3D printed with high temperature resin on a Form 2 stereolithography 3D printer (Formlabs, United States). Following 3D printing moulds were cured for 30 min with UV light at 60°C. The moulds were then autoclaved and used to cast cuboidal channels in molten 4% (w/v) agarose (Fig. S1A). Next an MSC laden hydrogel was cast into the channels which was composed of 60x10^6^ passage 4 MSCs/mL 4% (w/v) oxidised alginate (OA) prepared in XPAN as previously described [43]. The casted OA hydrogel was then crosslinked with 60 mM CaCl_2_ for 45 min at 37°C. Hence, a 2% (w/v) agarose (50°C) lid was added on top of the hydrogel forming an upper confinement. Casted hydrogels were cultured for 3 weeks in chondrogenic media (CDM+) composed of DMEM GlutaMAX supplemented with 100 U/mL penicillin, 100 μg/mL of streptomycin (both Biosciences, Ireland), 100 μg/mL of sodium pyruvate, 40 μg/mL L-proline, 50 μg/mL L-ascorbic acid-2-phosphate, and 1.5 mg/mL of bovine serum albumin, 50 μg/mL L-ascorbic acid-2-phosphate, 100 nM of dexamethasone, 4.7 μg/mL of linoleic acid (all Sigma, Ireland), 1X insulin-transferrin-selenium (Biosciences, Ireland), and 10 ng/mL of human transforming growth factor-β3 (TGF- β3) (PeproTech, USA).

### 2.3 Methacrylated xanthan gum preparation

The XGMA bioprinting support bath was prepared following previously published protocols [26,27,44]. In brief, 0.5 g of xanthan gum was dissolved in 100 mL of ultrapure water. Glycidyl methacrylate (4 mL) was then added to the solution and incubated at 60°C for 12 h with constant stirring. The resulting mixture was dialyzed using a 6-8 kDa molecular weight cutoff membrane for one week at room temperature in the dark. After dialysis, the solution was freeze-dried and stored at −20°C until further use. For bioprinting applications, XGMA (1% (w/v)) was reconstituted in phenol red–free DMEM supplemented with 100 U/mL penicillin, 100 μg/mL streptomycin, and 2.5 μg/mL amphotericin B. Lithium phenyl-2,4,6-trimethylbenzoylphosphinate (0.25% (w/v)) was used as the photoinitiator.

### 2.4 3D bioprinting

For 3D bioprinting a syringe pump printhead (CELLINK, Sweden) was used on the CELLINK BIO X6 (CELLINK, Sweden) with an extrusion rate of 1 μL/s for all experiments. The cell-only bioink (no supporting ink) was prepared by centrifuging passage 4 MSCs and gently loading the cells into a 3 mL syringe barrel (BD Plastipak scientific laboratory supplies, Ireland) with a 2.5 mL reduced dead space syringe plunger (Unifix UKMEDI, UK) with a blunt 25-gauge needle. Different filament widths were 3D bioprinted by adjusting bioprinting speed (5-15 mm/s). After bioprinting the XGMA bath was UV crosslinked (12 mW/cm^2^) for a duration of 4 min and is not removed during culture. The bioprinted filaments of varying widths were cultured for 21 days in CDM+ with complete media changes every 2-3 days. Next, filaments were bioprinted with a constant speed of 15 mm/s and cultured in CDM + supplemented with verteporfin (1 μM) YAP-inhibitor or Y-27632 (10 μM) Rock inhibitor (both MedChemTronica, Sweden) and controls with no inhibitors. Filaments were cultured for 21 days with complete media changes every 2-3 days where inhibitors were refreshed at each media change. Afterwards MSCs were bioprinted into a two-layered sheet (5 mm by 3 mm) in the XY plane and cultured for 28 days in CDM+. Finally, MSCs were bioprinted into circumferential lines to mimic the circumferential fibres of native meniscus and cultured for 28 days in CDM+ with complete media changes every 2-3 days. At the end of culture and prior to histological and biochemical assays of all bioprinted samples XGMA was physically removed from bioprinted samples through careful mechanical disruption using a scalpel and tweezer.

### 2.5 Live/dead imaging

Cell viability was assessed using the Live/Dead© assay kit (Invitrogen, Bioscience). Bioprinted filaments were first rinsed twice with PBS. A staining solution was then prepared containing 2 μM calcein and 4 μM ethidium homodimer-1 (EthD-1). Next the filaments were incubated at 37°C for 1 h in the staining solution. Following incubation, the filaments were rinsed twice with PBS and imaged immediately using a Leica SP8 confocal microscope. Calcein fluorescence was detected with excitation at 485 nm and emission at 530 nm, while EthD-1 was measured using excitation at 530 nm and emission at 645 nm. Maximum-intensity projections of z-stack images were generated to evaluate cell viability across the entire thickness of the filaments.

### 2.6 Histological evaluation

Histology samples were first embedded in 2% (w/v) agarose and then fixed with 4% paraformaldehyde at 4°C for 12 h. After fixation, samples were gradually dehydrated in ethanol solutions (50% - 100%), cleared in xylene, and finally paraffin wax embedded (all Sigma, Ireland). Samples were sliced into 5 μm sections with a microtome (Leica Microsystems, Ireland). Slides were stained with hematoxylin and eosin (H&E), alcian blue (AB) (1% (w/v), pH 1) with nuclear fast red counterstain to visualize sulfated glycosaminoglycans (sGAG), picrosirius red (PR) (0.1% (w/v)) for collagen, and alizarin red (AR) (1% (w/v), pH 4.1) for calcium deposition (all from Sigma, Ireland). To evaluate collagen organization PR-stained slides were imaged using polarized light microscopy (PLM). On ImageJ collagen fibre orientation and coherency were calculated. For each sample PLM quantification was performed across several section depths.

### 2.7 Immunohistochemistry analysis

As previously described [45,46], to determine the collagen types produced, immunohistochemistry using diaminobenzidine (DAB) was performed for type I collagen (Abcam, ab90395, 1:400) and type II collagen (Santa Cruz, sc52658, 1:400). Staining was visualized using a DAB substrate kit (Vector Labs).

### 2.8 Immunofluorescence analysis

Immunofluorescence staining was performed for YAP and F-actin as previously described [27,47]. Briefly, samples were incubated with YAP primary antibody (Abcam ab52771 1:250) followed by the secondary (Donkey Anti-Rabbit IgG H&L (Alexa Fluor® 488) ab150073 1:200) and then a DAPI (Sigma Aldrich, D9542 1:1000) incubation for 10 min. For F-actin (Alexa Fluor 488 phalloidin, ThermoFisher A12379 1:40) samples were stained for 30 min at room temperature followed by a DAPI (Sigma Aldrich, D9542 1:1000) incubation for 10 min. On ImageJ F-actin and cell nuclei orientation and coherency were measured.

### 2.9 Biochemical analysis

Samples were digested overnight at 60°C in a papain solution (3.88 U/mL) of pH 6.5 containing 0.1 M sodium acetate, 5 mM L-cysteine–hydrochloride hydrate, 5 mM ethylenediaminetetraacetic acid (EDTA) (all from Sigma, Ireland) under continuous rotation of 40 RPM. DNA was quantified using the Quant-iT™ PicoGreen® dsDNA Kit (Molecular Probes, Biosciences), and sulfated glycosaminoglycans (sGAGs) were measured with 1,9-Dimethyl-Methylene blue (pH 1.5, Sigma, Ireland) using chondroitin sulfate as a standard (Blyscan, Biocolor Ltd., UK). Excitation was read at 530 and 590 nm using the Synergy HT multi-detection micro-plate reader (BioTek Instruments, Inc). Total collagen content was determined by a hydroxyproline assay, with digested samples hydrolysed in 38% hydrochloric acid at 110°C for 18 h, dried, resuspended in ultrapure water, and then mixed with 2.82% (w/v) Chloramine T and 0.05% (w/v) 4-(dimethylamino)benzaldehyde (Sigma). A trans-4-hydroxy-L-proline standard (Fluka Analytical) was used, and absorbance was measured at 570 nm using a Synergy HT microplate reader (BioTek Instruments, Inc.). Finally, the total collagen content was calculated using the hydroxyproline-to-collagen ratio of 1:7.69 [48].

### 2.10 Mechanical testing

After 4 weeks of *in vitro* maturation, uniaxial tensile testing of 3D bioprinted sheets was performed using a 23 N load cell on a tensile biaxial tester (CellScale). Tests were performed at 37°C in a PBS bath. A 0.01 N preload was applied, followed by 10% strain held for 10 min to reach equilibrium, after which dynamic testing was performed over five cycles at 1Hz. The ramp modulus was determined from the linear portion of the stress-strain curves, the equilibrium modulus was calculated from the equilibrium force, and the dynamic modulus was derived from the force amplitude and strain, averaged across all five cycles.

### 2.11 Brillouin microscopy

Brillouin microscopy was performed to assess the micromechanical properties of the 3D bioprinted MSC filaments. Measurements were acquired using an inverted Brillouin microscope (Discoverer, CellSense Technologies GmbH, Berlin, Germany) equipped with a 780 nm laser and a 20X air objective (Olympus, NA 0.50, UPlanFI). Brillouin images were acquired using a lateral step size of 5 μm. MSCs filaments cultured for 10 days under YAP inhibition, ROCK inhibition, or control conditions (no inhibitor) were gently transferred to glass-bottom petri dishes fitted with #1.5 coverslips and imaged under hydrated conditions. All imaging was performed without XGMA removal. Importantly, Brillouin data in this study were acquired at a focal plane of 100 μm inside the filament to minimize contributions from the surrounding bath. Extraction and analysis of the Brillouin frequency shifts were performed using BMicro (https://github.com/BrillouinMicroscopy/BMicro, version 0.11.0). For quantitative comparison, the Brillouin frequency shift values averaged over all pixels within a filament interior ROI, and each data point in the plots corresponds to the ROI-averaged value from one repeated scan.

### 2.12 Statistical analysis

Statistical analyses were performed using analysis of variance (ANOVA) with Tukey's multiple comparisons in GraphPad Prism (GraphPad Software, CA, USA). Data are presented as mean ± standard deviation, and significance was accepted at p < 0.05.

## 3 Results

### 3.1 External geometric constraints can direct neotissue collagen in casted and bioprinted MSC filaments

Prior to bioprinting, an MSC laden oxidised alginate (OA) hydrogel was cast in non-adherent agarose channels (width = 500 μm, Fig. S1A and B) to determine whether external physical boundaries can direct neotissue collagen alignment. Over a 3-week culture the OA degraded, allowing the MSCs to self-organize into aggregates within the channels (Fig. S1C). The MSCs formed a robust tissue within the channels, with an ECM rich in sGAG and collagen as evident by the positive alcian blue and picrosirius red staining (Fig. S1D). There was also some calcium deposition observed, as seen in the alizarin red staining (Fig. S1D). PLM imaging indicated a preferential collagen alignment along the long axis of the agarose boundary (Fig. S1E and F).

Having demonstrated that physical boundaries can direct collagen alignment, we next sought to determine if 3D bioprinting could be used to control the extent of physical constraint applied to MSCs. To this end we bioprinted MSCs into a XGMA support bath at different bioprinting speeds to generate filaments with varying widths (250, 580, and 1000 μm) (Fig. 1A). Live/Dead imaging performed at day 7 revealed a cell viability higher than 80% in all filament widths (Fig. 1B and C). Following 3 weeks of culture in chondrogenic media, the bioprinted MSCs had formed handleable filaments that could be physically removed from the XGMA support bath (Fig. 2A). Histological analysis confirmed positive chondrogenesis, with robust sGAG and collagen deposition (Fig. 2A). PLM analysis revealed that neotissue collagen was preferentially aligned along the long axis (0°) of the bioprinted filaments in all conditions as evident in the color map images (Fig. 2B). However, only the thinnest filaments (250 μm) exhibited collagen alignment that was consistent and continuous throughout the full depth of the tissue (Fig. 2B). In contrast, thicker filaments only displayed collagen alignment along the edges of the filaments with no alignment in the core (Fig. 2B). Biochemical quantification revealed that total DNA increased with increasing filament width, consistent with the greater volume in wider filaments (Fig. 2C). When normalized to DNA, sGAG deposition was highest in the thinnest filaments, indicating enhanced chondrogenic matrix production under tighter confinement. Collagen/DNA content was greatest in the intermediate (580 μm) filaments, which could potentially be linked to differences in nutrient transport and/or paracrine signalling in the different groups. For subsequent bioprinting experiments the 250 μm width was chosen since it supported more homogenous collagen alignment throughout the filament.

**Fig. 1:**
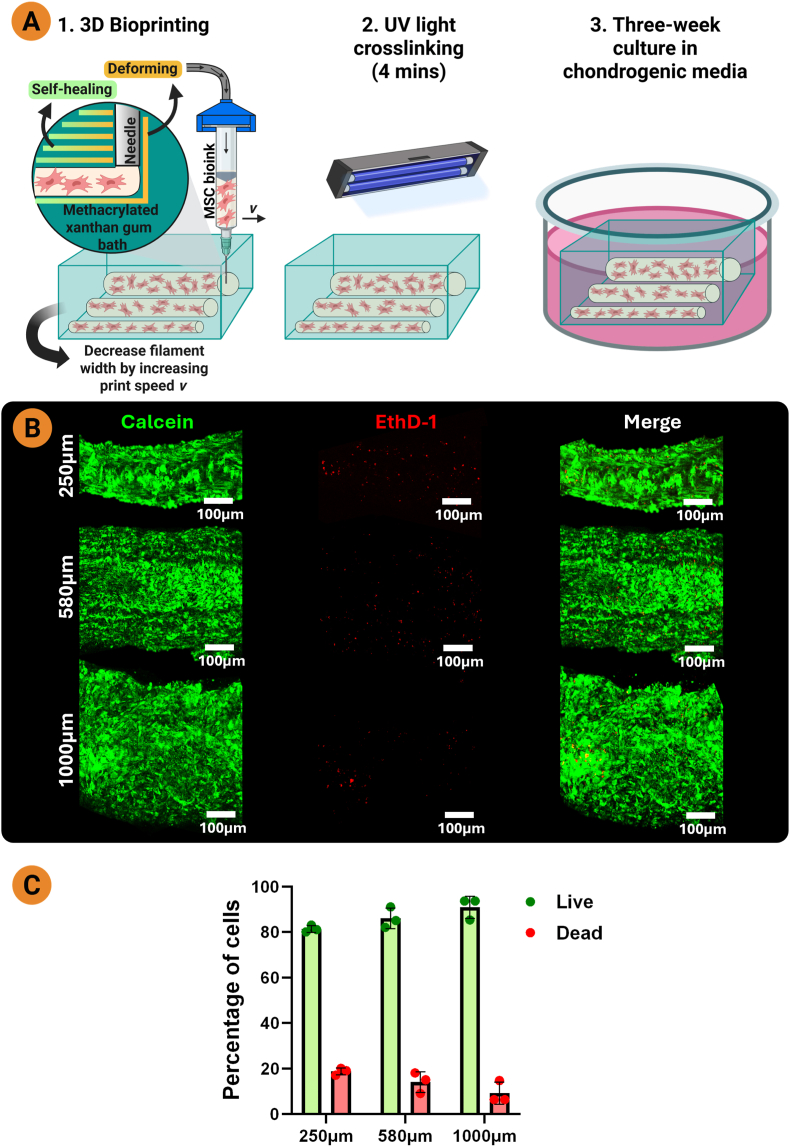
All filament widths support high cell viability. A) Diagrammatic representation of 3D MSC-only 3D bioprinting in XGMA support bath. Filament width is controlled by adjusting bioprinting speed. B) Live/Dead imaging on day 7 of bioprinted filaments (n = 3). C) Live/Dead quantification (n = 3). Created with biorender.com.

**Fig. 2:**
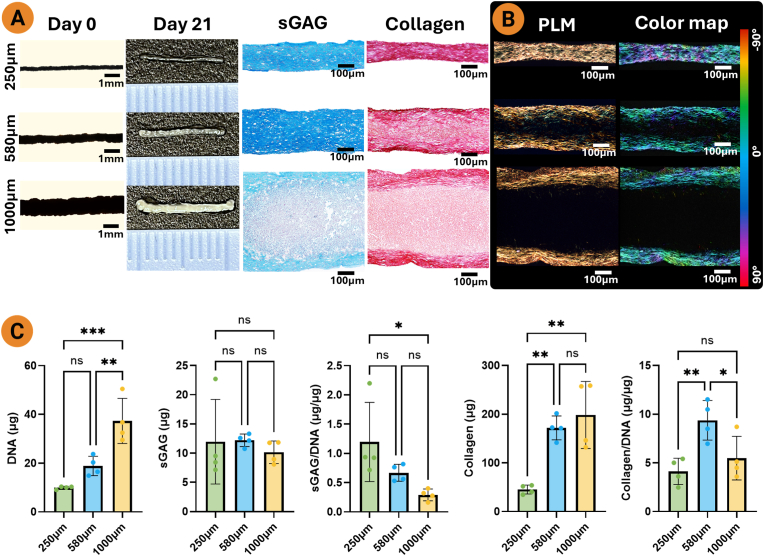
XGMA functioned as a boundary directing neotissue collagen along the long axis of bioprinted filaments. A) Macroscopic images of bioprinted filaments, histological staining with alcian blue (sGAG) and picrosirius red (collagen). B) PLM imaging and color map analysis of collagen. C) Biochemical quantification of DNA, sGAG, and collagen. An ordinary one-way analysis of variance (ANOVA) with Tukey's multiple comparisons was used for statistical analysis (n = 4). Significance levels: ns = not significant, ∗p < 0.05, ∗∗p < 0.01, ∗∗∗p < 0.001. (For interpretation of the references to color in this figure legend, the reader is referred to the Web version of this article.)

### 3.2 Neotissue collagen alignment is maintained following YAP and ROCK inhibition

**Fig. 3:**
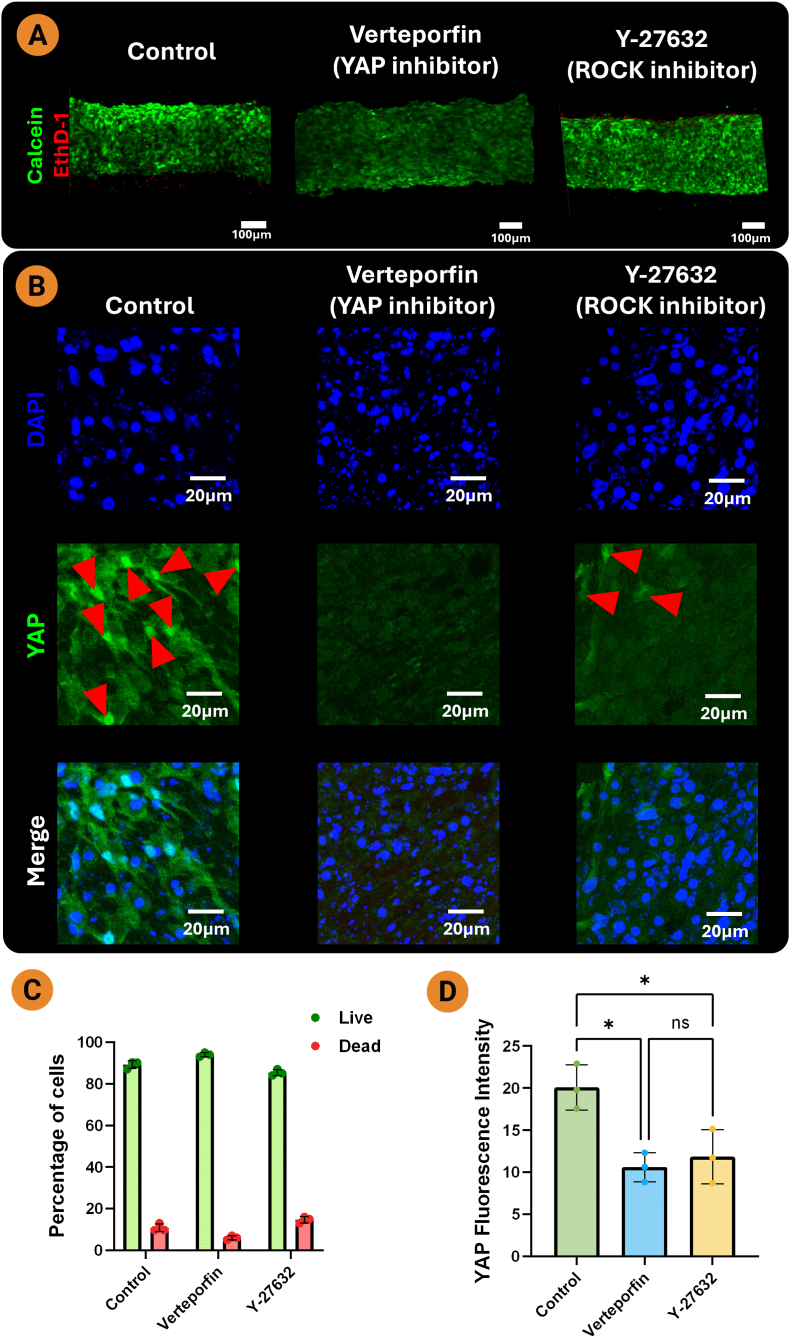
A) Live/Dead imaging on day 7 of bioprinted filaments treated with YAP or ROCK inhibitor and control. B) Immunofluorescence staining of YAP and DAPI at day 21 of bioprinted filaments treated with YAP or ROCK inhibitor and control (n = 3) with positive YAP nuclear localization in control filaments. C) Live/Dead quantification (n = 3). D) Quantification of YAP immunofluorescence intensity. An ordinary one-way analysis of variance (ANOVA) with Tukey's multiple comparisons was used for statistical analysis (n = 3). Significance levels: ns = not significant, ∗p < 0.05.

To determine whether the observed collagen alignment resulted from cell-medicated contractile forces or primarily from the geometric confinement imposed by the external boundaries, we investigated the role of key mechanotransduction pathways on tissue development. Specifically, the YAP or ROCK pathways were continuously inhibited in bioprinted filaments (250 μm width) over a 21-day culture in chondrogenic media using Verteporfin or Y-27632, respectively. Untreated filaments with no inhibitors were also included as controls. The viability of MSCs was not significantly affected by the inhibitor treatments with a viability greater than 80% in all groups (Fig. 3A, C). We next performed YAP immunofluorescence to investigate the activity of YAP in the different groups. Confocal imaging showed positive nuclear YAP staining in the control group (Fig. 3B, red arrows) of the bioprinted filaments, with a weak stain in the ROCK inhibitor group (Fig. 3B, red arrows). No nuclear YAP was observed in the YAP inhibitor group, confirming its inhibition. Additionally, the control group had a significantly higher nuclear YAP immunofluorescence intensity than both inhibitor groups (Fig. 3B, D).

**Fig. 4:**
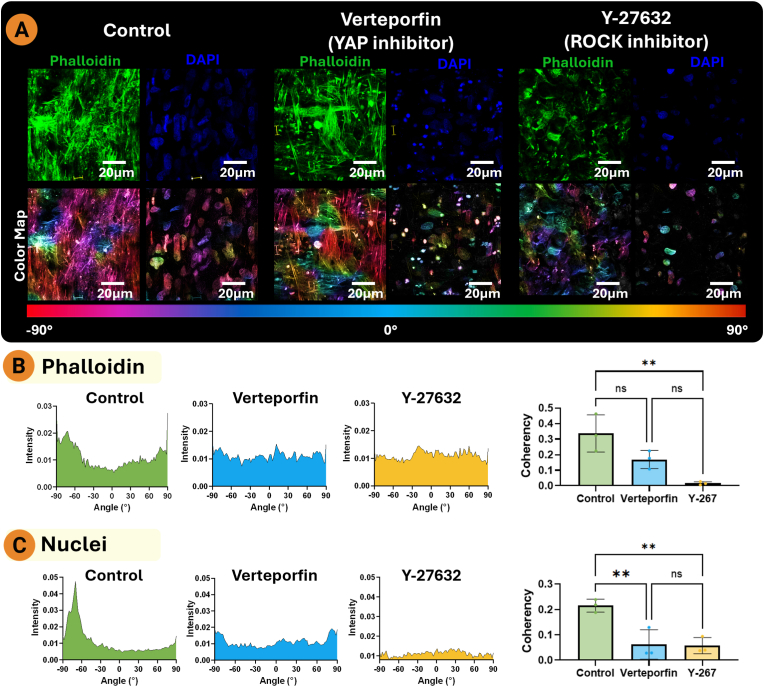
A) Immunofluorescence staining of F-actin and DAPI at day 14 of bioprinted filaments treated with YAP or ROCK inhibitors and control. Color map analysis of F-actin and nuclei orientation. Intensity and coherency graphs for B) phalloidin and C) DAPI. An ordinary one-way analysis of variance (ANOVA) with Tukey's multiple comparisons was used for statistical analysis (n = 3). Significance levels: ns = not significant, ∗∗p < 0.01. (For interpretation of the references to color in this figure legend, the reader is referred to the Web version of this article.)

We also sought to investigate whether geometric confinement induced any effect on cellular and nuclear alignment. Phalloidin staining of the actin cytoskeleton demonstrated pronounced cell alignment parallel to the long axis of the boundary in the control group, whereas this observed alignment was disrupted in both inhibitor groups (Fig. 4A and B). Reduced phalloidin staining was observed in the ROCK inhibitor group. Similarly, DAPI nuclear staining showed strong nuclei alignment in the control filaments. In the YAP inhibitor group, nuclei were somewhat aligned with shallow peaks evident at 90 and -90° (Fig. 4A, C). No preferential direction of alignment for the nuclei was observed in the ROCK inhibitor group (Fig. 4A, C). Quantitative coherency analysis confirmed that both the cytoskeletal and nuclear alignment were highest in the control group (Fig. 4B and C).

**Fig. 5:**
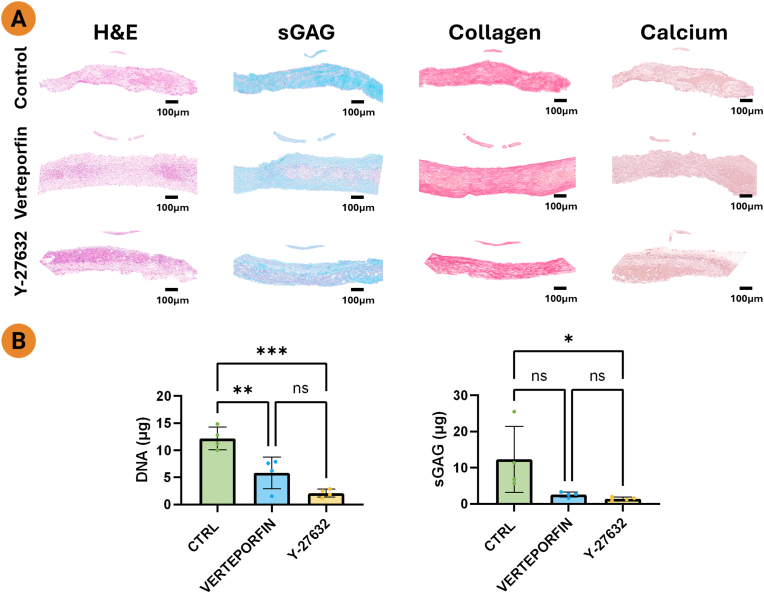
A) Histological staining with hematoxylin and eosin (H&E), alcian blue (sGAG), picrosirius red (collagen), alizarin red (calcium). B) Biochemical quantification of DNA, sGAG, and collagen. An ordinary one-way analysis of variance (ANOVA) with Tukey's multiple comparisons was used for statistical analysis (n = 4). Significance levels: ns = not significant, ∗p < 0.05, ∗∗p < 0.01, ∗∗∗p < 0.001. (For interpretation of the references to color in this figure legend, the reader is referred to the Web version of this article.)

**Fig. 6:**
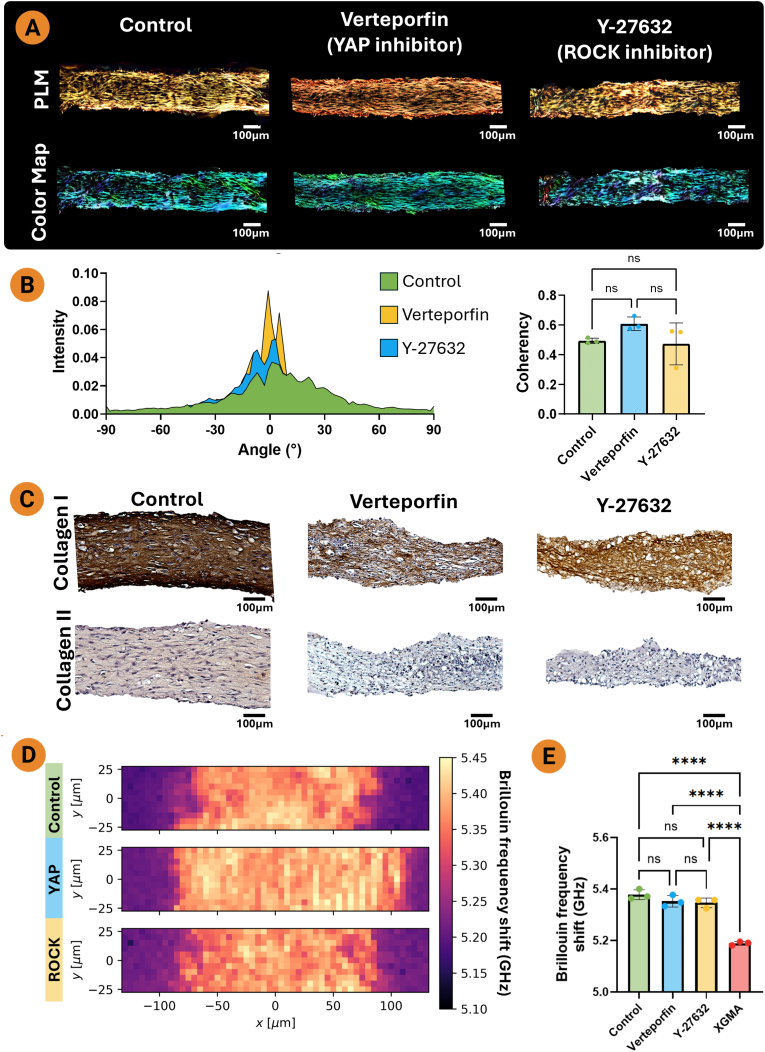
Collagen alignment along the long axis of physical boundary is maintained despite YAP and ROCK inhibition. A) PLM imaging and color map analysis of collagen. B) Collagen intensity and coherency graphs. C) Immunohistochemistry for collagens I and II. Nuclei stained with hematoxylin. D) Heat map of Brillouin frequency shift. E) Brillouin frequency shift measurement. An ordinary one-way analysis of variance (ANOVA) with Tukey's multiple comparisons was used for statistical analysis (n = 3). Significance levels: ns = not significant, ∗∗∗∗p < 0.0001. (For interpretation of the references to color in this figure legend, the reader is referred to the Web version of this article.)

Following inhibition of YAP and ROCK pathways in bioprinted filaments, histological staining at day 21 confirmed positive sGAG and collagen deposition in all filaments (Fig. 5A). Biochemical quantification revealed the control group had greater sGAG deposition (Fig. 5B). PLM color map analysis showed neotissue collagen was still preferentially aligned parallel to the long axis of the boundary for all bioprinted filaments (Fig. 6A). This was further confirmed with orientation distribution analysis with a peak at 0° in the intensity graph for all groups (Fig. 6B). No differences between the inhibitor groups and the control were observed for collagen fibre coherency (Fig. 6B). Coherency is a measure of the variance of collagen fibre distribution, where the closer the value is to one the lower the variance. PLM color map analysis was also performed on the filaments on days 7 and 14, which reveals temporal changes in collagen alignment, where at day 7 there is an initial alignment against the boundary wall, while at day 14 alignment is observed throughout the width of the filament (Fig. S2). Immunohistochemical staining demonstrated a fibrocartilage-like phenotype, with positive type I and weak type II collagen staining observed in all groups (Fig. 6C). No differences in Brillouin frequency shift were observed (Fig. 6D and E), suggesting no relative differences in Brillouin-derived micromechanical tissue properties.

### 3.3 XGMA support bath functions as an external boundary to direct a circumferential collagen organization in bioprinted fibrocartilage

**Fig. 7:**
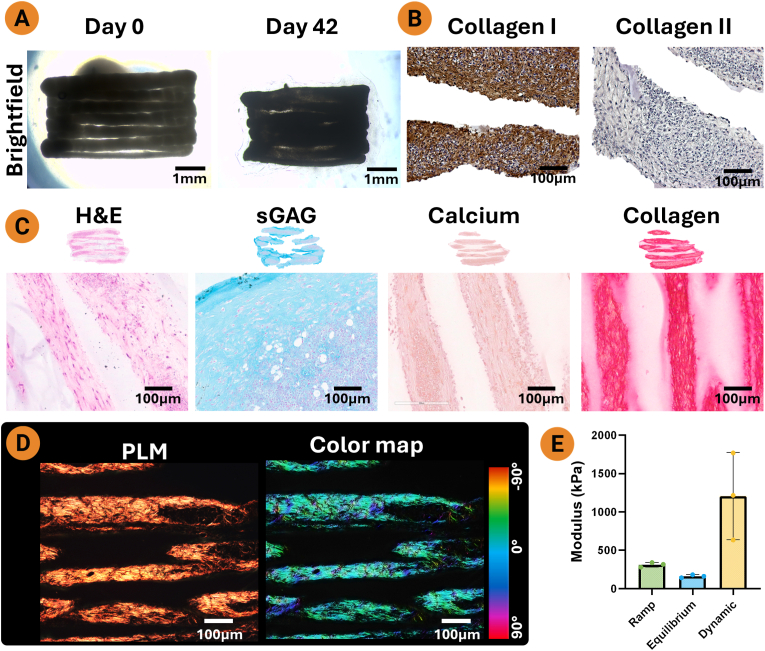
A) Brightfield images of bioprinted MSC sheet. B) Immunohistochemistry for collagens I and II. Nuclei stained with hematoxylin. C) Histological staining with hematoxylin and eosin (H&E), alcian blue (sGAG), picrosirius red (collagen), alizarin red (calcium). D) PLM imaging and color map analysis of collagen. E) Ramp, equilibrium and dynamic moduli measured during uniaxial tensile testing (n = 3). (For interpretation of the references to color in this figure legend, the reader is referred to the Web version of this article.)

**Fig. 8:**
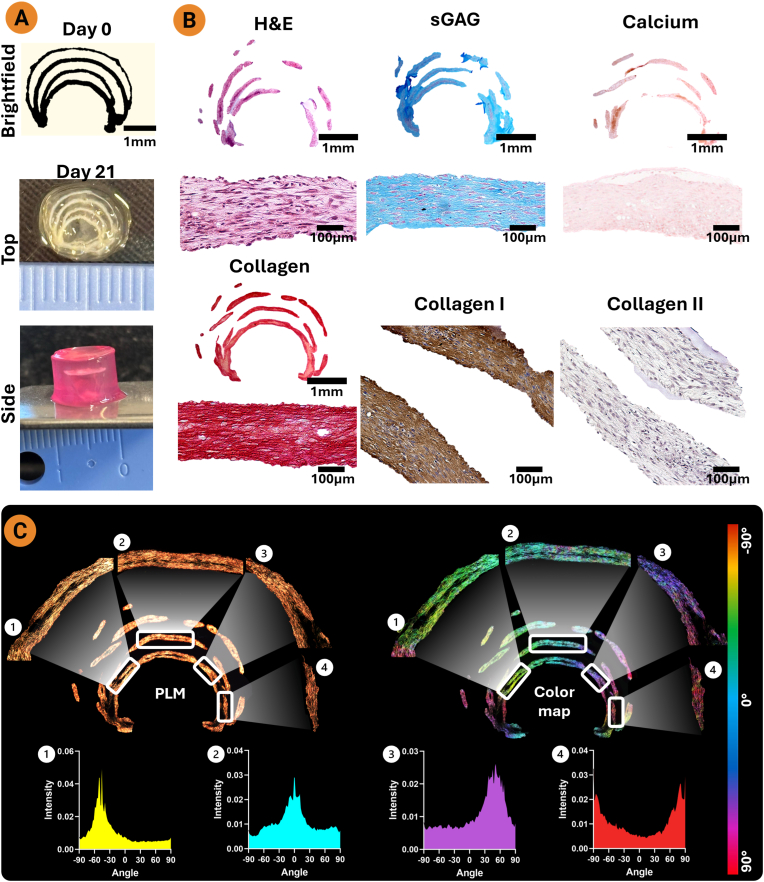
A) Brightfield image of bioprinted meniscal-like construct at day 0. Top and side views of bioprint at day 21. B) Histological staining with hematoxylin and eosin (H&E), alcian blue (sGAG), picrosirius red (collagen), alizarin red (calcium). Immunohistochemistry for collagens I and II. C) PLM imaging and color map analysis of collagen. Collagen intensity graphs (n = 3). (For interpretation of the references to color in this figure legend, the reader is referred to the Web version of this article.)

To translate these findings to a scaled-up and clinically relevant size, 250 μm wide filaments of MSCs were adjacently 3D bioprinted in XGMA forming a rectangular sheet (5 mm by 3 mm) in effort to engineer an anisotropic fibrocartilage tissue and cultured for 6 weeks in chondrogenic media (Fig. 7A). 250 μm wide filaments were used in the bioprinted sheet as it resulted in superior collagen alignment when compared to wider filaments (Fig. 2B). Fibrochondrogenesis was confirmed by positive sGAG and collagen staining (Fig. 7C), with immunohistochemistry revealing the development of a tissue that was rich in type I but not type II collagen (Fig. 7B). PLM colour map analysis indicated collagen alignment along the long axis of the bioprinted sheet (Fig. 7D). Tensile mechanical testing was used to measure the ramp, equilibrium and dynamic moduli of the engineered sheet which were found to be 311.2, 161.1 and 1206.3 kPa, respectively (Fig. 7E). The equilibrium modulus measures the contribution of the solid phase of the construct to its mechanical properties when no fluid flow is occurring. In contrast, during dynamic testing significant fluid pressurization occurs, leading to the higher reported dynamic modulus values. Subsequently, a more complex meniscus-shaped construct was bioprinted composed of concentric circumferential lines of MSCs in a XGMA support bath (Fig. 8A). Following 3 weeks of culture, the engineered tissue stained positive for sGAG and collagen while being negative for calcium deposits (Fig. 8B). A more intense type I compared to type II collagen stain confirmed a fibrocartilaginous phenotype (Fig. 8B). Importantly, PLM analysis showed that the collagen within the resulting neotissue followed the bioprinting path, resulting in a circumferential biomimetic alignment (Fig. 8C).

## 4 Discussion

Herein it was demonstrated that external physical boundaries can help direct collagen alignment within the tissue generated using MSC-only bioinks. We first demonstrated that casted MSCs in nonadherent cuboidal agarose channels (500 μm width) will self-organize and align their ECM along the long axis of their geometric constraint. This is in agreement with previous studies where physical boundaries have been shown to function as guiding structures for encapsulated cells [24,26]. These findings demonstrate that collagen alignment can emerge in the absence of cell adhesive cues, highlighting the role of geometry as a fundamental organizational cue. A limitation of this study is the lack of true unconstrained controls, which would be technically difficult to realise, therefore we cannot completely exclude the possibility that collagen would spontaneously organize in the absence of our physical boundaries. Building on our initial findings in agarose channels, we next bioprinted MSCs into a XGMA support bath which functioned to provide physical boundaries during *in vitro* culture. By adjusting bioprinting speed 3 different filament widths were bioprinted, namely 250, 580 and 1000 μm. The thinner width supported superior collagen alignment throughout the depth of the tissue along the long axis of the filament. This is consistent with previous studies using articular cartilage progenitor cell and MSC microtissue based bioinks bioprinted in XGMA [26,27], where the support bath not only enabled high resolution bioprinting but also functioned as a physical boundary to direct neotissue collagen alignment. Interestingly a previous study that casted MSCs into a wider range of channel widths found enhanced cell and collagen alignment in narrower 25-100 μm wide channels compared to 500-1000 μm wide channels [49]. The fact that we used a higher cell density while also confining our cells from the top could explain why we still observed collagen alignment in wider channel widths. Higher cell densities likely lead to increases in tissue growth and swelling (e.g. due to the production of negatively charged sGAGs), which may subsequently impact collagen organization.

It is well known that cells can sense and respond to their external physical environment through activation of various signalling pathways. For example, YAP and ROCK are key mediators in mechanotransduction that regulate cytoskeletal organization, remodelling, cell morphology, force generation and ultimately phenotype in response to a cell's external environment [[32], [33], [34], [35], [36]]. Thus, we sought to decouple the effects of cell generated forces from the geometrical cues on the observed neotissue collagen alignment in our thinnest bioprinted MSC filaments (250 μm) where the greatest alignment was seen. Verteporfin and Y-27632 were used to inhibit YAP and ROCK, respectively, in the bioprinted filaments for up to 21 days *in vitro*. YAP inhibition was confirmed with YAP immunofluorescence staining, revealing no nuclear YAP localization following inhibition. In agreement with other studies, a reduction in nuclear YAP localization was also observed in the ROCK inhibitor group [32,50]. ROCK inhibition was confirmed with loss and disorganization of cell stress fibres [50,51]. Inhibition of both YAP and ROCK pathways disrupted cell and nuclear alignment within our bioprinted filaments, which can be linked to their role as mediators of cell and nuclear deformation [[50], [51], [52], [53], [54]]. Despite suppression of YAP or ROCK, neotissue collagen alignment was maintained in bioprinted filaments, with no differences in collagen organization coherency when compared to untreated filaments. This suggests that collagen alignment is not dependent on cell and nuclei alignment. These findings suggest that geometric constraints can directly regulate collagen organization independent of cell-mediated processes. However, it is important to note that only two mechanotransduction pathways were investigated in this study and that other YAP or ROCK independent mechanosensitive signalling pathways may have contributed to the observed collagen alignment. This could include, for example, a role for ERK signalling which can still be activated in MSCs under ROCK inhibition [50].

Next, the bioprinted filaments treated with and without YAP or ROCK inhibitors were evaluated with Brillouin imaging. Brillouin microscopy is a label-free, non-invasive technique for assessing the three-dimensional viscoelastic properties of biological tissues, in which the Brillouin frequency shift depends on the material's elastic properties [[55], [56], [57]]. Notably, neither YAP nor ROCK inhibition altered the Brillouin frequency shift when compared to the control group (no inhibitor), suggesting no change in the micromechanical properties of the tissue that formed in the bioprinted filaments, which shows consistency since no differences were observed in the collagen alignment.

Recapitulating the circumferential collagen architecture seen in the native meniscus is a key goal in the field of meniscus bioprinting and tissue engineering [[23], [24], [25],^58^]. Previous studies have used synthetic polymers such as polycaprolactone (PCL) to create physical boundaries for bioprinted cells, or have explored microfibre bioink additives to guide cell and collagen alignment [24,25]. PCL has a relatively slow degradation rate and is known to cause fibrosis *in vivo* [59,60], which may limit its clinical applications. While microfibre bioink additives represents a promising approach, challenges include clogging during extrusion and the persistence of microfibres within the final graft. Cell only bioprinting is an attractive approach as it could enable the engineering of scaffold-free grafts free of materials that may trigger a foreign body response *in vivo*. Previous work with MSC only bioprinting for meniscal engineering has shown promise, with ‘C’ shaped constructs positive for sGAG deposition following *in vitro* culture in chondrogenic media [61,62]. However, these studies did not assess collagen phenotype or organization in the engineered grafts, which are key determinants of tissue functionality. In this study we scaled up from MSC only bioprinting of individual filaments and bioprinted an anisotropic sheet-like fibrocartilage tissue with collagen alignment parallel to the long axis of the sheet. Building on this a tissue with a circumferentially organised collagen architecture was engineered by leveraging circumferential XGMA physical cues. This highlights that the physical boundaries still functioned as geometric cues when scaled up to larger tissues. It is important to note that although the tensile mechanical properties of the bioprinted sheet are approaching the MPa range, they are still dramatically lower than the native meniscus [63], which can be linked to the overall levels of collagen synthesis and cross-linking within these bioprinted constructs. In the future additional characterization of the anisotropic mechanical properties is required to fully assess the mechanical functionality of the engineered tissue.

Future work will focus on controlled degradation of XGMA bioprinting support bath to enable for the engineering of truly scaffold-free meniscal grafts by employing a temporally supportive bioprinting platform. It is important to note that while we were able to engineer anisotropic fibrocartilage tissue using this approach, the resulting grafts do not fully recapitulate the complex collagen network of the native meniscus. Additionally, in this study we did not focus on recapitulating the wedge shape nor the inner and outer regions of the native meniscus. The use of a spatial biochemical cues within the bath could be a promising approach to achieve a heterogenous phenotype within the engineered graft, with a type II collagen rich inner region and a type I collagen rich outer region as in native meniscus. Another limitation of the study is the somewhat limited characterization of the extracellular matrix of our engineered tissues. In the future the expression of other zonal meniscal gene and protein markers such as COMP, decorin and biglycan should also be evaluated, and more detailed comparisons to the native tissue (in terms to total matrix accumulation, phenotype etc) should be undertaken. A potential concern with the proposed approach is that within larger multi-layered constructs, nutrient diffusion limitations and hypoxic regions may occur. However, one benefit of 3D bioprinting is the control over internal graft architecture; previous studies have shown that the incorporation of nutrient channels coupled with a bioreactor culture can facilitate the development of scaled-up grafts and similar approaches could be envisioned for our bioprinted constructs [64]. Additionally, it may also be possible to locally release glucose which is known to be a key metabolite for maintaining cell viability [65]. This can be addressed with the use of oxygen and glucose releasing materials which could potentially be incorporated in the support bath itself [66,67]. Finally, we believe the concept of boundary guided collagen alignment can be translated and applied to engineer other musculoskeletal tissues such as ligaments and tendons where achieving a biomimetic collagen alignment is critical to graft functionality.

## 5 Conclusions

This study demonstrates that external geometric confinement is an important cue for directing neotissue collagen alignment in engineered fibrocartilage. Using both casted and embedded bioprinted MSC constructs, we show that physical boundaries alone can induce robust, preferential collagen organization along the axis of confinement. Inhibition of YAP and ROCK disrupted cellular and nuclear alignment but did not impair collagen organization, suggesting that boundary-induced collagen alignment might occur independent of cell-generated forces, however other mechanosensitive signalling pathways not explored here may have contributed to collagen network development. Leveraging the principle of boundary induced alignment, we successfully engineered structurally anisotropic fibrocartilage sheets and circumferentially aligned meniscus-like constructs using an XGMA support bath. Together, these findings demonstrate that embedded bioprinting not only enables high-resolution biofabrication, but can also be strategically leveraged to introduce geometric cues in a maturing graft, providing a powerful platform for engineering meniscal grafts with a more biomimetic collagen architecture.

## CRediT authorship contribution statement

**Aliaa Sherif Karam:** Conceptualization, Data curation, Formal analysis, Investigation, Methodology, Supervision, Validation, Visualization, Writing – original draft, Writing – review & editing. **Gabriela S. Kronemberger:** Conceptualization, Investigation, Methodology, Supervision, Validation, Writing – review & editing. **Kaoutar Chattahy:** Investigation, Methodology, Writing – review & editing. **Diana Eveline Sanchez-Amador:** Data curation, Formal analysis, Investigation, Visualization, Writing – review & editing. **Michael G. Monaghan:** Conceptualization, Funding acquisition, Resources, Writing – review & editing. **Daniel J. Kelly:** Conceptualization, Funding acquisition, Methodology, Project administration, Resources, Supervision, Writing – review & editing.

## Funding

This work was funded by the European Research Council (ERC, 4D-BOUNDARIES 101019344) and funding from the ERC under the European Union's Horizon 2020 research and innovation programme (grant agreement ERC-2023-CoG-101125153) and the Research Ireland Frontiers for the Future award (Grant No.22/FFP-P/11394)

## Declaration of competing interest

The authors declare that they have no known competing financial interests or personal relationships that could have appeared to influence the work reported in this paper.

## Figures and Tables

**figs1:**
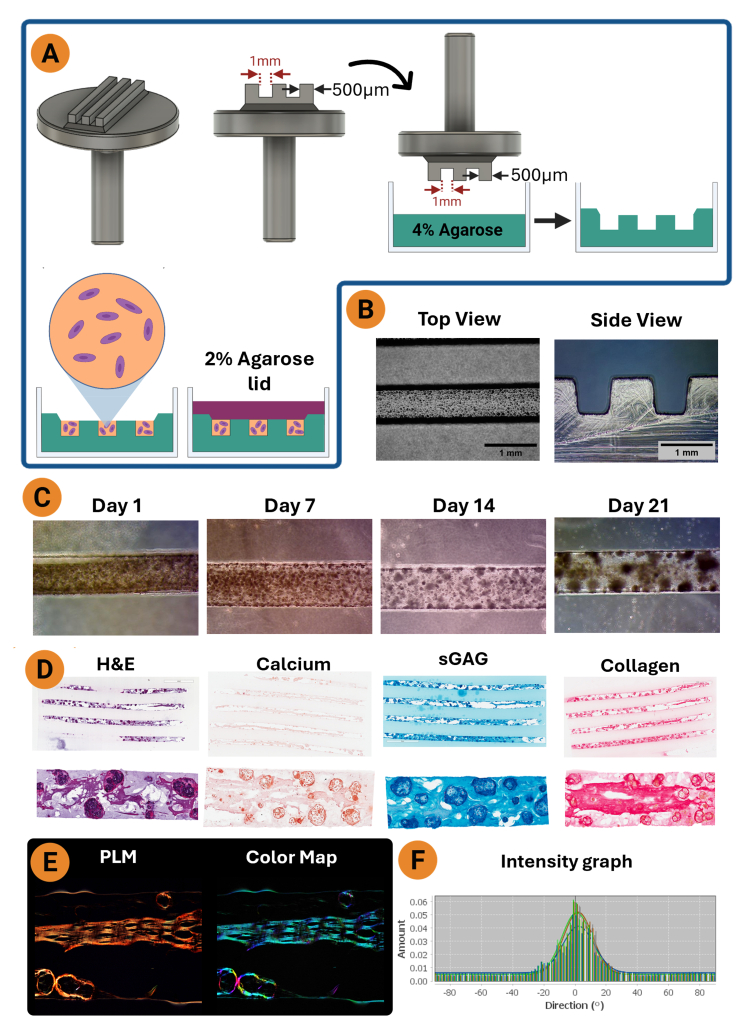


**figs2:**
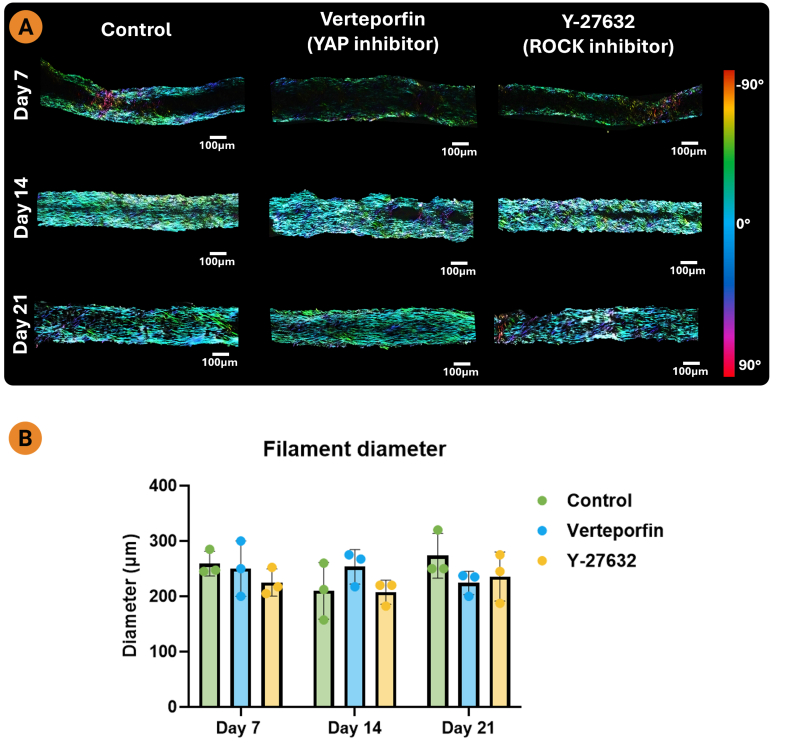


## Data Availability

Data will be made available on request.

## References

[1] Gannon A.R., Nagel T., Bell A.P., Avery N.C., Kelly D.J. (2015). Postnatal changes to the mechanical properties of articular cartilage are driven by the evolution of its collagen network. Eur. Cell. Mater..

[2] Fox A.J., Wanivenhaus F., Burge A.J., Warren R.F., Rodeo S.A. (2015 Mar). The human meniscus: a review of anatomy, function, injury, and advances in treatment. Clin. Anat..

[3] Bullough P.G., Munuera L., Murphy J. (1970). The strength of themenisci of the knee as it relates to their fine structure. J BoneJoint Surg Br.

[4] Florescu S., Zaharia C., Drăghici G.A., Nica D.V., Damian C.G. (2025). The impact of aging on meniscal tears and chondral lesions in men: insights from first-time arthroscopic. Knee Evaluation. Life (Basel, Switzerland).

[5] Englund M., Guermazi A., Gale D., Hunter D.J., Aliabadi P., Clancy M., Felson D.T. (2008). Incidental meniscal findings on knee MRI in middle-aged and elderly persons. N. Engl. J. Med..

[6] Heijink A., Gomoll A.H., Madry H., Drobnič M., Filardo G., Espregueira-Mendes J., Van Dijk C.N. (2012 Mar). Biomechanical considerations in the pathogenesis of osteoarthritis of the knee. Knee Surg. Sports Traumatol. Arthrosc..

[7] Englund M., Guermazi A., Roemer F.W., Aliabadi P., Yang M., Lewis C.E., Torner J., Nevitt M.C., Sack B., Felson D.T. (2009). Meniscal tear in knees without surgery and the development of radiographic osteoarthritis among middle-aged and elderly persons: the multicenter osteoarthritis study. Arthritis Rheum..

[8] Englund M., Roos E.M., Lohmander L.S. (2003). Impact of type of meniscal tear on radiographic and symptomatic knee osteoarthritis: a sixteen-year followup of meniscectomy with matched controls. Arthritis Rheum..

[9] Pengas I.P., Assiotis A., Nash W., Hatcher J., Banks J., McNicholas M.J. (2012). Total meniscectomy in adolescents: a 40-year follow-up. J. Bone Jt. Surg. Br. Vol..

[10] Wang Y., Shepherd J. (2025). Collagen-based scaffolds for meniscal repair and regeneration. J. Tissue Eng. Regen. Med..

[11] Song P., Chen H., Ma H., Zhou Y., Zhang Y. (2026). Integrated strategies in meniscus tissue engineering: from biomaterials to stem cell-driven regeneration. Front. Bioeng. Biotechnol..

[12] Baker B.M., Gee A.O., Sheth N.P., Huffman G.R., Sennett B.J., Schaer T.P., Mauck R.L. (2009). Meniscus tissue engineering on the nanoscale: from basic principles to clinical application. J. Knee Surg..

[13] Salazar A.K., Brown J.L. (2025). Tissue engineering approaches to recapitulate the Micro- and macro-architecture of the knee meniscus. Regener. Eng. Transl. Med..

[14] Shimomura K., Hamamoto S., Hart D.A., Yoshikawa H., Nakamura N. (2018). Meniscal repair and regeneration: current strategies and future perspectives. J. Clin. Orthop.Trauma.

[15] Figueroa F., Figueroa D., Calvo R., Vaisman A., Espregueira-Mendes J. (2019). Meniscus allograft transplantation: indications, techniques and outcomes. EFORT Open Rev..

[16] Trentacosta N., Graham W.C., Gersoff W.K. (2016). Meniscal allograft transplantation: state of the art. Sports Med. Arthrosc. Rev..

[17] Dienst M., Greis P.E., Ellis B.J., Bachus K.N., Burks R.T. (2007). Effect of lateral meniscal allograft sizing on contact mechanics of the lateral tibial plateau: an experimental study in human cadaveric knee joints. Am. J. Sports Med..

[18] Fritz J., Moser A.C., Otahal A., Redl H., Teuschl-Woller A.H., Schneider K.H., Nehrer S. (2025). Silk fibroin-based hydrogels supplemented with decellularized extracellular matrix and gelatin facilitate 3D bioprinting for meniscus tissue engineering. Macromol. Biosci..

[19] Lian L., Xie M., Luo Z., Zhang Z., Maharjan S., Mu X., Garciamendez-Mijares C.E., Kuang X., Sahoo J.K., Tang G., Li G., Wang D., Guo J., González F.Z., Abril Manjarrez Rivera V., Cai L., Mei X., Kaplan D.L., Zhang Y.S. (2024). Rapid volumetric bioprinting of decellularized extracellular matrix bioinks. Adv. Mater..

[20] Stocco T.D., Moreira Silva M.C., Corat M.A.F., Gonçalves Lima G., Lobo A.O. (2022). Towards bioinspired meniscus-regenerative scaffolds: engineering a novel 3D bioprinted patient-specific construct reinforced by biomimetically aligned nanofibers. Int. J. Nanomed..

[21] Lan X., Ma Z., Szojka A.R.A., Kunze M., Mulet-Sierra A., Vyhlidal M.J., Boluk Y., Adesida A.B. (2021). TEMPO-oxidized cellulose nanofiber-alginate hydrogel as a bioink for human meniscus tissue engineering. Front. Bioeng. Biotechnol..

[22] Filardo G., Petretta M., Cavallo C., Roseti L., Durante S., Albisinni U., Grigolo B. (2019). Patient-specific meniscus prototype based on 3D bioprinting of human cell-laden scaffold. Bone Joint Res..

[23] Chae S., Lee S.S., Choi Y.J., Hong D.H., Gao G., Wang J.H., Cho D.W. (2021). 3D cell-printing of biocompatible and functional meniscus constructs using meniscus-derived bioink. Biomaterials.

[24] Barceló X., Eichholz K., Gonçalves I., Garcia O., Kelly D. (2023). Bioprinting of structurally organized meniscal tissue within anisotropic melt electrowritten scaffolds. Acta Biomater..

[25] Prendergast M.E., Heo S.J., Mauck R.L., Burdick J.A. (2023). Suspension bath bioprinting and maturation of anisotropic meniscal constructs. Biofabrication.

[26] Karam A.S., Kronemberger G.S., Chattahy K., Kelly D.J. (2025). Cell-only bioprinting of articular cartilage progenitor cells within a physically constraining support Bath to engineer structurally organized grafts. Bioact. Mater..

[27] Spagnuolo F.D., Kronemberger G.S., Kelly D.J. (2025). A 4D bioprinting platform to engineer anisotropic musculoskeletal tissues by spatially patterning microtissues into temporally adapting support baths. bioRxiv.

[28] Saeidi N., Karmelek K.P., Paten J.A., Zareian R., DiMasi E., Ruberti J.W. (2012). Molecular crowding of collagen: a pathway to produce highly-organized collagenous structures. Biomaterials.

[29] Nerger B.A., Brun P.T., Nelson C.M. (2019). Microextrusion printing cell-laden networks of type I collagen with patterned fiber alignment and geometry. Soft Matter.

[30] Casale C., Imparato G., Mazio C., Netti P.A., Urciuolo F. (2021). Geometrical confinement controls cell, ECM and vascular network alignment during the morphogenesis of 3D bioengineered human connective tissues. Acta Biomater..

[31] Saeidi N., Sander E.A., Zareian R., Ruberti J.W. (2011). Production of highly aligned collagen lamellae by combining shear force and thin film confinement. Acta Biomater..

[32] Dupont S., Morsut L., Aragona M., Enzo E., Giulitti S., Cordenonsi M., Zanconato F., Le Digabel J., Forcato M., Bicciato S., Elvassore N., Piccolo S. (2011). Role of YAP/TAZ in mechanotransduction. Nature.

[33] Panciera T., Azzolin L., Cordenonsi M., Piccolo S. (2017). Mechanobiology of YAP and TAZ in physiology and disease. Nat. Rev. Mol. Cell Biol..

[34] Kim E., Riehl B.D., Bouzid T., Yang R., Duan B., Donahue H.J., Lim J.Y. (2024). YAP mechanotransduction under cyclic mechanical stretch loading for mesenchymal stem cell osteogenesis is regulated by ROCK. Front. Bioeng. Biotechnol..

[35] Andalib M.N., Lee J.S., Ha L., Dzenis Y., Lim J.Y. (2013). The role of RhoA kinase (ROCK) in cell alignment on nanofibers. Acta Biomater..

[36] Guan G., Cannon R.D., Coates D.E., Mei L. (2023). Effect of the Rho-Kinase/ROCK signaling pathway on cytoskeleton components. Genes.

[37] Calvo F., Ege N., Grande-Garcia A., Hooper S., Jenkins R.P., Chaudhry S.I., Harrington K., Williamson P., Moeendarbary E., Charras G., Sahai E. (2013). Mechanotransduction and YAP-dependent matrix remodelling is required for the generation and maintenance of cancer-associated fibroblasts. Nat. Cell Biol..

[38] Wyckoff J.B., Pinner S.E., Gschmeissner S., Condeelis J.S., Sahai E. (2006). ROCK- and myosin-dependent matrix deformation enables protease-independent tumor-cell invasion in vivo. Curr. Biol..

[39] Amano M., Ito M., Kimura K., Fukata Y., Chihara K., Nakano T., Matsuura Y., Kaibuchi K. (1996). Phosphorylation and activation of myosin by Rho-associated kinase (Rho-kinase). J. Biol. Chem..

[40] Kimura K., Ito M., Amano M., Chihara K., Fukata Y., Nakafuku M., Yamamori B., Feng J., Nakano T., Okawa K., Iwamatsu A., Kaibuchi K. (1996). Regulation of myosin phosphatase by Rho and Rho-associated kinase (Rho-kinase). Science (New York, N.Y.).

[41] Riento K., Ridley A.J. (2003). Rocks: multifunctional kinases in cell behaviour. Nat. Rev. Mol. Cell Biol..

[42] Tominaga T., Barber D.L. (1998). Na-H exchange acts downstream of RhoA to regulate integrin-induced cell adhesion and spreading. Mol. Biol. Cell.

[43] Barceló X., Eichholz K., Garcia O., Kelly D. (2022). Tuning the degradation rate of alginate-based bioinks for bioprinting functional cartilage tissue. Biomedicines.

[44] Patrício S., Sousa L., Correia T. (2020). Freeform 3D printing using a continuous viscoelastic supporting matrix. Biofabrication.

[45] Browe D.C., Díaz-Payno P.J., Freeman F.E., Schipani R., Burdis R., Ahern D.P., Nulty J.M., Guler S., Randall L.D., Buckley C.T., Brama P.A.J., Kelly D.J. (2022). Bilayered extracellular matrix derived scaffolds with anisotropic pore architecture guide tissue organization during osteochondral defect repair. Acta Biomater..

[46] Barceló X., Garcia O., Kelly D. (2023). Chondroitinase ABC treatment improves the organization and mechanics of 3D bioprinted meniscal tissue. ACS Biomater. Sci. Eng..

[47] Eichholz K.F., Freeman F.E., Pitacco P., Nulty J., Ahern D., Burdis R., Browe D.C., Garcia O., Hoey D.A., Kelly D.J. (2022). Scaffold microarchitecture regulates angiogenesis and the regeneration of large bone defects. Biofabrication.

[48] Ignat’eva N., Danilov N., Averkiev S., Obrezkova M., Lunin V., Sobol E. (2007). Determination of hydroxyproline in tissues and the evaluation of the collagen content of the tissues. J. Anal. Chem..

[49] Chou C.L., Rivera A.L., Sakai T., Caplan A.I., Goldberg V.M., Welter J.F., Baskaran H. (2013 May). Micrometer scale guidance of mesenchymal stem cells to form structurally oriented cartilage extracellular matrix. Tissue Eng Part A.

[50] Driscoll T.P., Cosgrove B.D., Heo S.J., Shurden Z.E., Mauck R.L. (2015). Cytoskeletal to nuclear strain transfer regulates YAP signaling in mesenchymal stem cells. Biophys. J..

[51] Coleman P.R., Lay A.J., Ting K.K., Zhao Y., Li J., Jarrah S., Vadas M.A., Gamble J.R. (2020). YAP and the RhoC regulator ARHGAP18, are required to mediate flow-dependent endothelial cell alignment. Cell Commun. Signal. : CCS.

[52] Xu K., Sun F., Hu Y., Hou N., Wang S., Huang C. (2025). Deciphering the contribution of ROCK-dependent actin cytoskeleton remodeling to testosterone production in mouse leydig cells. Cells.

[53] Landau S., Ben-Shaul S., Levenberg S. (2018). Oscillatory strain promotes vessel stabilization and alignment through fibroblast YAP-mediated mechanosensitivity. Adv. Sci..

[54] Maekawa M., Ishizaki T., Boku S., Watanabe N., Fujita A., Iwamatsu A., Obinata T., Ohashi K., Mizuno K., Narumiya S. (1999). Signaling from Rho to the actin cytoskeleton through protein kinases ROCK and LIM-kinase. Science (New York, N.Y.).

[55] Prevedel R. (2019). Brillouin microscopy: an emerging tool for mechanobiology. Nat. Methods.

[56] Bailey M., Correa N., Harding S., Stone N., Brasselet S., Palombo F. (2020). Brillouin microspectroscopy data of tissue-mimicking gelatin hydrogels. Data Brief.

[57] Kabakova I., Zhang J., Xiang Y., Caponi S., Bilenca A., Guck J., Scarcelli G. (2024). Brillouin microscopy. Nat. Rev. Methods Primers.

[58] Jian Z., Zhuang T., Qinyu T., Liqing P., Kun L., Xujiang L., Diaodiao W., Zhen Y., Shuangpeng J., Xiang S., Jingxiang H., Shuyun L., Libo H., Peifu T., Qi Y., Quanyi G. (2020). 3D bioprinting of a biomimetic meniscal scaffold for application in tissue engineering. Bioact. Mater..

[59] Lam C., Hutmacher D., Schantz J., Woodruff M., Teoh S. (2009). Evaluation of polycaprolactone scaffold degradation for 6 months in vitro and in vivo. J. Biomed. Mater. Res..

[60] Sommerfeld S., Cherry C., Schwab R. (2019). Interleukin-36γ-producing macrophages drive IL-17-mediated fibrosis. Sci. Immunol..

[61] Ding A., Lee S.J., Tang R., Gasvoda K.L., He F., Alsberg E. (2022). 4D cell-condensate bioprinting. Small.

[62] Jeon O., Lee Y.B., Jeong H., Lee S.J., Wells D., Alsberg E. (2019). Individual cell-only bioink and photocurable supporting medium for 3D printing and generation of engineered tissues with complex geometries. Mater. Horiz..

[63] Henderson B.S., Cudworth K.F., Wale M.E., Siegel D.N., Lujan T.J. (2022). Tensile fatigue strength and endurance limit of human meniscus. J. Mech. Behav. Biomed. Mater..

[64] Daly A.C., Sathy B.N., Kelly D.J. (2018). Engineering large cartilage tissues using dynamic bioreactor culture at defined oxygen conditions. J. Tissue Eng..

[65] Gurian M., Allijn I.E., Veenendaal L., Bassous N., Shin S.R., Leijten J. (2025). Self-feeding of engineered tissues via controlled glucose release facilitates survival and vascularization of living implants. Adv. Funct. Mater..

[66] Wang Z., Chen T., Li X., Guo B., Liu P., Zhu Z., Xu R.X. (2023 Aug 9). Oxygen-releasing biomaterials for regenerative medicine. J. Mater. Chem. B.

[67] Zargarzadeh M., Gomes M.C., Patrício S.G., Custódio C.A., Mano J.F. (2023). A self-sustaining hydrogels with autonomous supply of nutrients and bioactive domains for 3D cell culture. Adv. Funct. Mater..

